# Interfacial Properties of Anisotropic Monolayer SiAs Transistors

**DOI:** 10.3390/nano14030238

**Published:** 2024-01-23

**Authors:** Feihu Zou, Yao Cong, Weiqi Song, Haosong Liu, Yanan Li, Yifan Zhu, Yue Zhao, Yuanyuan Pan, Qiang Li

**Affiliations:** 1 College of Physics, Qingdao University, Qingdao 266071, China; 2 State Key Laboratory of Heavy Oil Processing, Institute of New Energy, College of Chemistry and Chemical Engineering, China University of Petroleum (East China), Qingdao 266580, China

**Keywords:** monolayer SiAs, Schottky contact, Ohmic contact, transistors

## Abstract

The newly prepared monolayer (ML) SiAs is expected to be a candidate channel material for next-generation nano-electronic devices in virtue of its proper bandgap, high carrier mobility, and anisotropic properties. The interfacial properties in ML SiAs field-effect transistors are comprehensively studied with electrodes (graphene, V_2_CO_2_, Au, Ag, and Cu) by using ab initio electronic structure calculations and quantum transport simulation. It is found that ML SiAs forms a weak van der Waals interaction with graphene and V_2_CO_2_, while it forms a strong interaction with bulk metals (Au, Ag, and Cu). Although ML SiAs has strong anisotropy, it is not reflected in the contact property. Based on the quantum transport simulation, ML SiAs forms *n*-type lateral Schottky contact with Au, Ag, and Cu electrodes with the Schottky barrier height (SBH) of 0.28 (0.27), 0.40 (0.47), and 0.45 (0.33) eV along the *a* (*b*) direction, respectively, while it forms *p*-type lateral Schottky contact with a graphene electrode with a SBH of 0.34 (0.28) eV. Fortunately, ML SiAs forms an ideal Ohmic contact with the V_2_CO_2_ electrode. This study not only gives a deep understanding of the interfacial properties of ML SiAs with electrodes but also provides a guide for the design of ML SiAs devices.

## 1. Introduction

The continuous down-scaling requirement of transistors has aroused intensive research enthusiasm in channel semiconductors in the past decades [1]. As the gate length gradually shrinks, traditional bulk silicon-based transistors face serious short channel effects, resulting in reduced gate control and increased leakage current [2]. Two-dimensional (2D) semiconductor materials are expected to be promising candidate channel materials for sub-10 nm metal–oxide–semiconductor field-effect transistors (MOSFET) in virtue of the atomic thickness and dangling bond-free smooth surface [2,3,4]. Thus, a tremendous research effort has been focused on the exploration of 2D semiconductors as the channel materials in MOSFETs, such as graphene [5], Bi_2_O_2_Se [6,7], and transition metal dichalcogenides (TMDs) [8,9,10], etc. Most of the studied 2D semiconductors have isotropous characteristics in planes with highly symmetrical crystal structures. Two-dimensional semiconductors with asymmetrical crystal structures possess anisotropic electronic and optical properties, etc., which brings a new degree of freedom in the plane [11,12,13,14,15,16]. Therefore, various advanced equipment related to angles is designed based on the 2D anisotropic semiconductors, such as photoelectric detectors, anisotropic memory, and gas sensors [17,18,19]. The development of anisotropic 2D semiconductors will open up a new path for the future generation of anisotropic polyfunctional electronic devices.

Binary IV–V group compounds (IV = Si, Ge and V = P, As), one class of 2D anisotropic semiconductors, have been successfully fabricated by high-pressure melt growth, mechanical exfoliation, and solid-source vapor phase growth [20,21,22,23]. Among them, SiAs possesses a band gap from 1.37 eV in bulk to 2.37 eV in monolayer (ML) [21,24]. The exfoliation energy of SiAs is 0.27 J m^−2^, which is smaller than that of graphene (0.32 J m*^−^*^2^), indicating the experimental feasibility of its monolayers [25,26]. At the same time, the predicted carrier mobility of ML SiAs is as high as 10^3^ cm^2^ V^−1^ s^−1^ [24]. What is more, the 2D SiAs field-effect transistor (FET) is fabricated in experiments in which the on/off ratio is 10^4^ and hole mobility anisotropy ratio is as high as 5.5 [21], which is even greater than the well-known 2D ReS_2_ (3.1) [27] and black phosphorene (1.5) [28]. These properties make 2D SiAs a promising candidate of channel materials for future transistors. However, due to the scarcity of suitable doping methods, 2D semiconductors are inevitably in direct contact with the electrode, and there is usually a Schottky barrier at the interface, which seriously affects the performance of the device [29,30]; the Schottky contact is also observed in 2D SiAs FET with a Au electrode [21]. The Schottky barrier cannot be tuned, on account of the strong Fermi-level pinning (FLP) effect when contacting bulk electrodes; thus, forming a low-barrier contact will be a huge challenge in 2D semiconductor FETs [31,32]. In contrast to the bulk metal electrodes, 2D electrodes are more appreciated, because a weak van der Waals (vdW) interaction between the 2D metal and the 2D semiconductor enables the tuning of Schottky barrier height (SBH) [33,34]. Therefore, the selection of 2D electrodes may improve the performance of the 2D SiAs transistor [35,36,37]. Whether the high anisotropy of ML SiAs affects the contact properties with metals has aroused our interest.

In this work, based on ab initio electronic structure calculations and quantum transport simulation, the interfacial characteristics in anisotropic ML SiAs FET with graphene, V_2_CO_2_, Au, Ag, and Cu electrodes are comprehensively researched for the first time. ML SiAs forms weak vdW interactions in contact with graphene and V_2_CO_2_, while it forms strong interactions in contact with bulk metals (Au, Ag and Cu), leading to the metallization of ML SiAs. ML SiAs FETs have the same contact type with electrodes along different transport directions (i.e., *a* and *b* directions). The *p*- or *n*-type lateral Schottky contacts are discovered in ML SiAs FETs with graphene, Au, Ag, and Cu electrodes, while an ideal Ohmic contact is discovered in ML SiAs FETs with the V_2_CO_2_ electrode. As we expected, the contact properties of ML SiAs with 2D electrodes are more excellent than that with bulk metal electrodes, especially for the V_2_CO_2_ electrode, which can form an ideal Ohmic contact in ML SiAs FET, and the Schottky barriers disappear at both the vertical and lateral interfaces.

## 2. Methodology

### 2.1. Interface and Device Models

**Figure 1:**
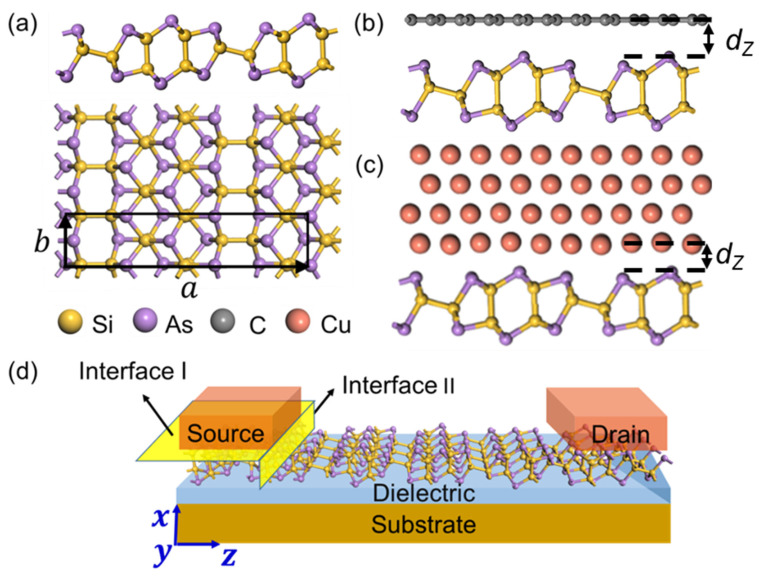
(**a**) Top and side views of the most stable atomic structures of ML SiAs. The black rectangle expresses the unit cell of ML SiAs. (**b**,**c**) Side view of ML SiAs on (**b**) graphene and (**c**) bulk metal surface. (**d**) Schematic illustration of ML SiAs FET. The yellow planes express the vertical (interface I) and the lateral (interface II) interfaces of ML SiAs FET. Yellow, purple, gray, and red balls express Si, As, C and Cu atoms, respectively.

Owing to good gate controllability and the smooth surface of 2D metal compared with that of bulk metal, 2D metals are also selected as electrodes for comparison. The lattice parameter of ML SiAs is fixed, while the lattice parameters of electrodes are changed to adapt to that of ML SiAs. To make the lattice mismatch less than 3%, graphene, V_2_CO_2_, Au, Ag, and Cu are chosen as electrodes. The 1 × 2 unit cells of ML SiAs match $\sqrt{7}\;\times \;2\sqrt{13}$ unit cells of Au (111), Ag (111), V_2_CO_2_, and $3\;\times \;5\sqrt{3}$ unit cells of Cu (111) and graphene with lattice mismatch ratios of 2.84%, 2.84%, 2.81%, 2.39%, and 0.15%, respectively. Four-layer atomic structures are used to emulate the surface of bulk metals, while single-layer structures are used to emulate the surface of the 2D metals, in which ML SiAs is adsorbed on a single side of the metal surfaces, as shown in Figure 1c. Since the interaction of ML SiAs with metals mainly exists in the surface atoms of the metal, two layers of metal atoms on the side away from ML SiAs are fixed, and all the other atoms are completely relaxed during the calculation.

A double-probe model is established to emulate ML SiAs FETs, as shown in Figure 1d. The fully optimized interfacial systems and the free-standing ML SiAs are used as electrodes and channels, respectively. Because of the highly anisotropic properties of ML SiAs, two different transport directions (*a* and *b* directions) are taken into consideration to explore the transport characteristics of the ML SiAs FET. The channel lengths of the ML SiAs FETs are set at 6.41 and 5.17 nm along the *a* and *b* directions, respectively.

### 2.2. Computational Methods

Density functional theory (DFT) is implemented to study the geometric structures and electrical properties of ML SiAs and the interfacial systems, which are performed in the Vienna ab initio simulation package (VASP) [38]. The generalized gradient approximation (GGA) with the Perdew−Burke−Ernzerhof (PBE) functional is used to deal with the exchange–correlation potential [39]. The projector-augmented wave (PAW) pseudopotential [40] and plane–wave basis set [41] are used, and the cut-off energy is 500 eV. The vdW interactions in interface systems are modified by the zero-damping DFT-D3 method of Grimme [42]. The energy converges to at least 1 × 10^−5^ eV/atom, and the force converges to at least 0.01 eV/Å. The *k*-points are selected at intervals of about 0.02 Å^−1^ in the Brillouin zone [43]. To avoid the interaction between the neighboring slabs, the height of the vacuum space is set to not less than 15 Å.

Quantum transport simulation is performed in the Atomistix Tool Kit (ATK) 2019 Package by combining DFT with non-equilibrium Green’s function (NEGF) [44]. The linear combination of the atomic orbitals (LCAO) base group is used in the calculation with the double-ζ plus polarization (DZP) form. Due to the influence of electrodes, the electron–electron Coulomb interaction in the channel region is basically shielded, so the single electron approximation can well describe the electronic behavior of FET [39]. It can be proved that the hole SBHs calculated at the DFT level (0.26/0.19/0.20 eV) of single/double/triple-layer phosphorene FETs with a Ni electrode are consistent with those extracted from the experiments (0.35/0.23/0.21 eV) [45,46,47]. Density mesh cut-off is 110 Hartree, and the temperature is 300 K. The boundary conditions along to the transport direction, perpendicular to the transport direction in the ML SiAs plane, and perpendicular to the transport direction out of the plane, are the Dirichlet, periodic, and Neumann types, respectively. The Monkhorst−Pack *k*-point meshes are 1 × 6 × 50 and 1 × 6 × 1 in the electrode region and central region, respectively. The transmission coefficient ($T^{k_{//}}\left(E\right)$) is derived by the formula:

(1) $$T^{k_{//}}\left(E\right)=\mathrm{Tr}\left[\Gamma_{L}^{k_{//}}\left(E\right)G^{k_{//}}\left(E\right)\Gamma_{R}^{k_{//}}\left(E\right)G^{k_{//}†}\left(E\right)\right]$$

in which *k*_//_ represents a reciprocal lattice vector point vertical to the transport direction. $G^{k_{//}}\left(E\right)/G^{k_{//}†}\left(E\right)\;$ present the retarded/advanced Green’s function. The level broadening $\Gamma_{L/R}^{k_{//}}\left(E\right)=i(\Sigma_{L/R}^{r,k_{//}}-\Sigma_{L/R}^{a,k_{//}})$ caused by the source and drain electrodes is represented by electrode self-energy $\Sigma_{L/R}^{k_{//}}$, which is used to depict the impression of electrodes on scattering area [48]. 

## 3. Results and Discussion

### 3.1. Binding Degree and Electronic Structure of the ML SiAs/Electrode Interfaces

The fully optimized atomic structure of ML SiAs is shown in Figure 1a. The black rectangle represents the unit cell of ML SiAs, which consists of 12 Si atoms and 12 As atoms. Each As atom coordinates with three Si atoms, and each Si atom coordinates with three As atoms and another Si atom. The relaxed lattice parameters of ML SiAs are *a* = 21.37 Å and *b* = 3.69 Å, which is consistent with the previous works [26,49]. Strong anisotropic lattice parameters of ML SiAs in the *a* and *b* directions will lead to diverse transport, electrical, optical, and thermal properties along the different in-plane directions, for example, the photocurrent anisotropy ratios of photodetectors fabricated with a single SiAs nanosheet at 514.5 nm as high as 5.3 in experiments [21].

**Figure 2:**
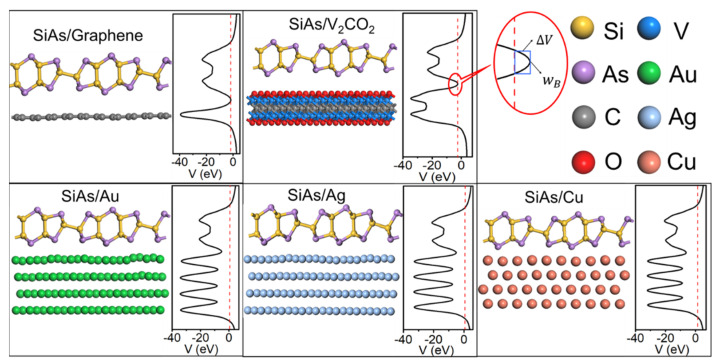
Side views of the most stable atomic structures and the average electrostatic potential distributions in the planes normal to the interfaces of ML SiAs on graphene, V_2_CO_2_, Au, Ag, and Cu surfaces. The enlargement is the electrostatic potential at the interface between SiAs and the electrode. The red dashed lines express the Fermi level.

The optimized atomic configurations of ML SiAs/electrode interfaces are shown in Figure 2. Compared with the atomic structures of the interfaces without optimization in Figure S1, the atomic configuration of ML SiAs is retained on all the electrode surfaces with a slight bond length change. The atomic configurations of graphene, V_2_CO_2_, and Cu electrodes are kept after optimization, while those of Au and Ag electrodes are slightly undulating. The complete data results of the ML SiAs/electrode interfaces are summarized in Table 1. The binding energy *E*_b_ between ML SiAs and electrodes is established by the following formula:

(2) $$E_{b}=(E_{\mathrm{SiAs}}+{\;E}_{M}-{\;E}_{\mathrm{SiAs-M}})/N$$

where *E*_SiAs_, *E*_M_, and *E*_SiAs-M_ represent the lowest energy of the free-standing ML SiAs, the pristine electrode surfaces, and the ML SiAs/electrode interfaces, respectively. *N* expresses the sum of the quantity of Si atoms and As atoms in the layer near the electrodes in each supercell. The binding energy *E*_b_ are 0.32, 0.46, 0.57, 0.59, and 0.74 eV/atom when ML SiAs contacts with graphene, V_2_CO_2_, Ag, Au, and Cu, respectively. The average distance ($d_{z}$) is determined as the average vertical distance between the top-most atoms of the metal surface and the plane of the As atom closest to the metal surface, as indicated in Figure 1c. The average distance $d_{z}$ between ML SiAs and Cu, Ag, Au, V_2_CO_2_, and graphene is 2.07, 2.30, 2.31, 2.55, and 3.29 Å, respectively. On the basis of the binding energy and the average distance, two types of ML SiAs/electrode interfaces are distinguished. The first class is ML SiAs/graphene and V_2_CO_2_ interfaces with small *E*_b_ (0.32 and 0.46 eV/atom) and large $d_{z}$ (2.55 and 3.29 Å), which is a typical vdW-type stacking feature, similar to ML BP ($d_{z}$ = 3.38 Å) and ML InSe ($d_{z}$ = 3.52 Å) on graphene [50,51]. The other is ML SiAs/Au, Ag, and Cu interfaces, which have strong interactions with large *E*_b_ (0.57~0.74 eV/atom) and small $d_{z}$ (2.07~2.31 Å), similar to ML BP (2.30 < $d_{z}$ < 2.44 Å and 0.43 < *E*_b_ < 0.59 eV/atom) and ML InSe (2.31 < $d_{z}$ < 2.66 Å and 0.72 < *E*_b_ < 0.86 eV/atom) on Au, Ag, and Cu surfaces [45,50,51].

**Figure 3:**
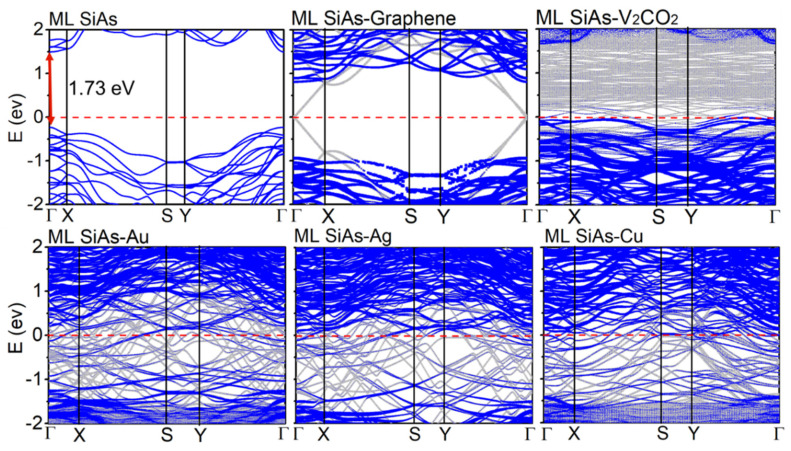
Band structures of the free-standing ML SiAs and ML SiAs/electrode interfaces (graphene, V_2_CO_2_, Au, Ag, and Cu). Gray and blue lines represent the band structure of the interfacial systems and projected to the ML SiAs, respectively. The red dashed lines express the Fermi level, which is set to zero.

The band structures of the free-standing ML SiAs and ML SiAs/electrode interfaces are shown in Figure 3. The conduction band minimum (CBM) and valence band maximum (VBM) of ML SiAs are both at the Γ point, indicating that it is a semiconductor with a direct band gap of 1.73 eV, which is well in agreement with the previous reports [26,49]. When ML SiAs is in contact with graphene and V_2_CO_2_, the band gap of ML SiAs/electrode interfaces are 1.73 eV and 1.70 eV, respectively, which are almost same as that of the pristine ML SiAs. Therefore, the band gap of ML SiAs is well retained due to the weak vdW interaction. The Fermi level of SiAs moves upward in contact with graphene accompanied by electron transfer from graphene to ML SiAs, while it moves downward in contact with V_2_CO_2_ accompanied by electron transfer from ML SiAs to V_2_CO_2_. In contrast, when ML SiAs is in contact with bulk metals (i.e., Au, Ag, and Cu), the band structure of ML SiAs is completely destructed, with the bandgaps disappearing, leading to the metallization of ML SiAs. The band structure of ML SiAs in the ML SiAs/electrode interfaces corresponds to the classification of the ML SiAs/electrode interfaces.

**Figure 4:**
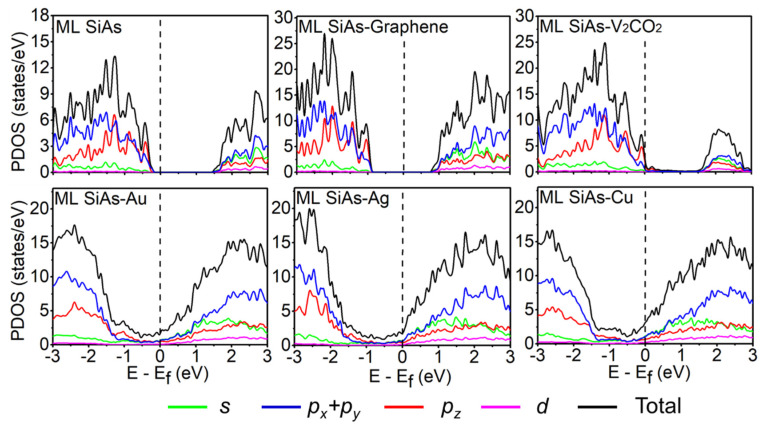
Projected density of states (PDOS) of the free-standing ML SiAs and ML SiAs on the graphene, V_2_CO_2_, Au, Ag, and Cu surfaces. The Fermi level is expressed by black dashed lines.

To acquire a better understanding of the electronic properties of the ML SiAs/electrode interfaces, the projected density of states (PDOS) of the pristine ML SiAs and the ML SiAs/electrode interfaces are also calculated, as shown in Figure 4. It is found that the electron state near the VBM of the free-standing ML SiAs is mainly originated from the hybridization of the *p* orbital of Si and As atoms, while that near the CBM is mostly originated from the *s* and *p* orbitals of Si and As atoms. For the weak bonding case, the bandgap of ML SiAs is kept in the ML SiAs/electrode interfaces with the shift of the Fermi level, which coincides with the result of the band structure. The contribution of the orbital to the CBM and the VBM is semblable to that in the pristine ML SiAs. For the strong bonding case (i.e., contacting with Au, Ag, and Cu), a large number of electron states expand into the bandgap of ML SiAs with the bandgap disappearing, which is a representative characteristic of metallization. The electron states near the Fermi level are mainly contributed by the *s* and *p* orbitals of ML SiAs in the strong bonding case. 

### 3.2. Schottky and Tunneling Barrier in ML SiAs Transistors

For 2D semiconductor FETs, it is unavoidable to create contact with the electrodes to regulate the polarity of the injected carriers. Thus, the tunneling and Schottky barrier at the 2D semiconductors/electrode interfaces will directly affect the performance of the FETs. When the electron or hole moves from the source/drain electrode to the channel in the ML SiAs FET, it will pass through two interfaces, as shown in Figure 1d. One is the interface I (vertical interface), which is along the vertical direction between the electrode and the ML SiAs under the electrode. The other is interface II (lateral interface), which is along the lateral direction between the interface system and the ML SiAs channel region. For the weak interacting interfacial system, a tunneling barrier possibly appears at the interface I, while the Schottky barrier possibly appears at the interfaces I and II. For the strong interacting interfacial system, there may be no barrier at the interface I due to the metallization of ML SiAs. Therefore, the ML SiAs/electrode interface is considered to be a new metal system, and a Schottky barrier may appear at the interface II. 

The tunneling barrier is determined to be the average electrostatic potential above the Fermi level at the interface I, as shown in Figure 2. In the strong bonding systems, the average electrostatic potentials are all lower than the Fermi level at the interfaces between ML SiAs and bulk metals (Au, Ag, and Cu), indicating that there are no tunneling barriers. However, the electrostatic potentials are slightly higher than the Fermi level at the ML SiAs/graphene and V_2_CO_2_ interfaces, so there are small tunneling barriers with heights of 0.02 and 0.15 eV, respectively. We assume that a square barrier is used instead of the real barrier to calculate the tunneling probability (*T*_B_). As illustrated in Figure 2, the barrier height and full width at half-maximum of the real barrier are selected as the barrier height (Δ*V*) and width (*w*_B_) of the square barrier, respectively. The tunneling probability *T*_B_ is estimated using the square barrier model, and the formula is as follows [52]:

(3) $$T_{B}=\exp(-2\frac{\sqrt{2m\Delta V}}{\hbar }\;\times \;w_{B})$$

where, *m* and ℏ are the mass of the free electron and the reduced Planck constant, respectively. The tunneling probabilities of ML SiAs/graphene and V_2_CO_2_ are 99.48% and 95.65%, respectively. The high tunneling probability means that electrons can easily tunnel at the interface I.

The Schottky barriers at the vertical interface are explored by the commonly used method, i.e., band structure calculation (BSC). The electron/hole SBH is extracted as the energy difference between the CBM/VBM of the ML SiAs and the Fermi energy level of the ML SiAs/electrode interfaces, which are extracted from the band structures of the ML SiAs/electrode interfaces, as shown in Figure 3 [53]. The Fermi level of the ML SiAs/graphene interface is located at the center of the bandgap and closer to the CBM of ML SiAs, indicating the formation of an *n*-type vertical Schottky contact with the electron SBH of 0.81 eV, while the Fermi level of the ML SiAs/V_2_CO_2_ interface is below the VBM of ML SiAs, indicating the formation of a *p*-type vertical Ohmic contact. Due to the metallization of ML SiAs, ML SiAs contacts with the bulk metal (Au, Ag, and Cu) to form a vertical Ohmic contact. In the BSC, the electrode region and channel region are regarded as separate parts; thus, the interaction between them is ignored. Particular attention needs to be paid when using the SBH acquired from the BSC.

**Figure 5:**
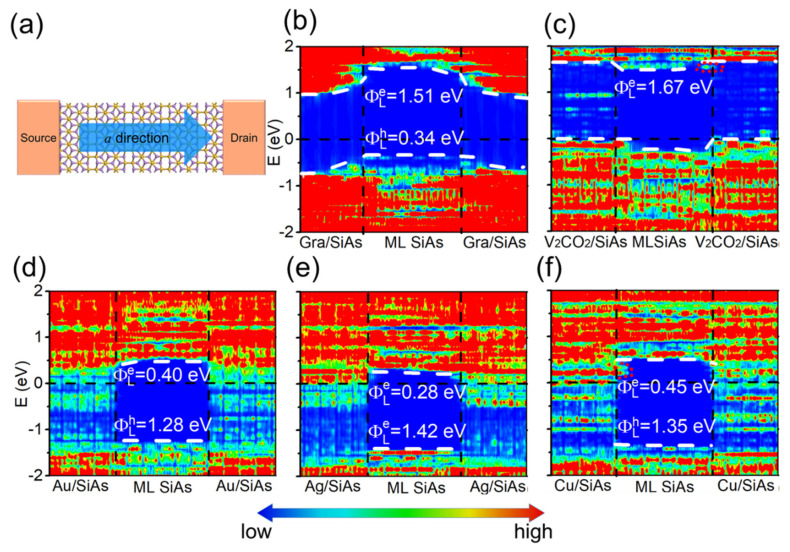
(**a**) Top view of the ML SiAs transistor with the transport direction along *a* direction. (**b**–**f**) Zero bias and zero gate voltage LDDOS (**left panel**) and transmission spectra (**right panel**) of the ML SiAs FETs with graphene, V_2_CO_2_, Ag, Au, and Cu electrodes. The horizontal black dashed lines express the Fermi level. The white dashed lines express the CBM/VBM of the ML SiAs. The red short dash lines represent metal-induced gap states in (**c**,**f**).

**Figure 6:**
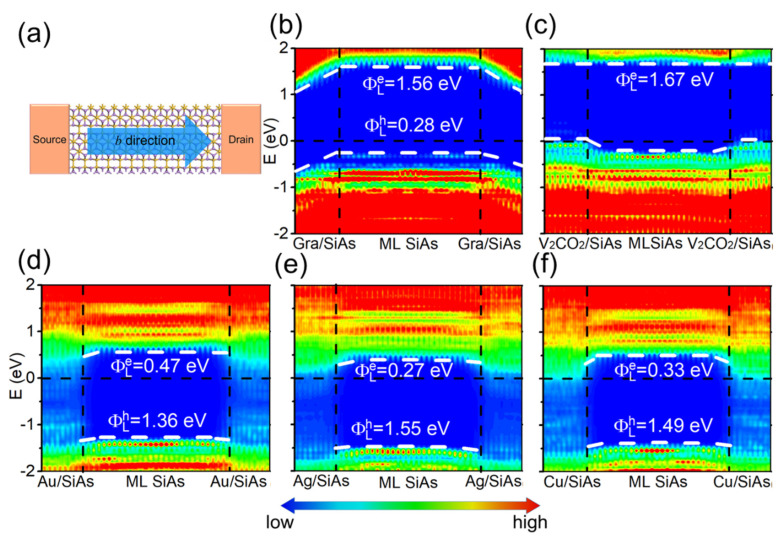
(**a**) Top view of the ML SiAs transistor with the transport direction along the *b* direction. (**b**–**f**) Zero bias and zero gate voltage LDDOS (**left panel**) and transmission spectra (**right panel**) of the ML SiAs FETs with graphene, V_2_CO_2_, Ag, Au and Cu electrodes. The horizontal black dashed lines express the Fermi level. The white dashed lines express the CBM/VBM of the ML SiAs.

According to previous reports [37,54,55], quantum transport simulation (QTS) can overcome the shortcoming of the BSC and is a more reliable method to examine SBH. It takes a double-probe model to simulate the transistor, taking an electrode and channel as a whole and considering the interaction between them. Therefore, we constructed a dual-probe model of the ML SiAs FETs to calculate the vertical SBH of the ML SiAs transistors, as shown in Figure 1d. Because of the strong anisotropy of ML SiAs, the transport properties along both the a and b directions are investigated. The local device density of states (LDDOS) of ML SiAs MOSFETs are calculated with different electrodes under zero bias voltage and zero gate voltage, as shown in Figure 5 and Figure 6.

On the basis of the LDDOS, the vertical electron/hole SBHs are extracted as the energy difference between the Fermi level and the CBM/VBM of ML SiAs at the edge of the electrode region away from the channel. The LDDOS of ML SiAs FETs along the *a* direction are revealed in Figure 5. The bandgap of ML SiAs is retained when graphene and V_2_CO_2_ are the electrodes. The ML SiAs/graphene contact forms a *p*-type vertical Schottky contact (hole SBH is 0.75 eV), while ML SiAs forms a vertical Ohmic contact with the V_2_CO_2_ electrode. Because the states of electron appear in the bandgap of ML SiAs in the electrode region, ML SiAs is hybridized with bulk metal electrodes (Au, Ag, and Cu), indicating the formation of vertical Ohmic contacts. The contact type and polarity at the vertical interfaces along the *b* direction (from the LDDOS in Figure 6) are the same as those along the *a* direction, except for the ML SiAs/graphene contact, where the vertical hole SBH along the *b* direction of 0.65 eV is lightly smaller than along the *a* direction of 0.75 eV. Thus, the strong anisotropy of ML SiAs is not reflected in the vertical contact. 

The contact type and polarity of the vertical barrier obtained by QTS are the same as those acquired from BSC, except for the graphene electrode. At the ML SiAs/graphene interface, the *n*-type Schottky contact in the BSC is transformed into the *p*-type Schottky contact in the QTS. The change of the contact polarity originates from the mutual effect between source/drain electrodes and the channel, which leads to the apparent band bending of ML SiAs at the electrode region with a graphene electrode, as shown in Figure 5b and Figure 6b. 

The lateral Schottky barriers are explored by the QTS, in which the lateral electron/hole SBH ($\Phi_{L,a}^{e}$/$\Phi_{L,a}^{h}$) is calculated by the energy difference between the Fermi level and the CBM/VBM of ML SiAs at the electrode/channel interface (i.e., around the vertical black dashed line in Figure 5b and Figure 6b) from the LDDOS. Along the *a* direction of ML SiAs FETs, it is found that the Fermi level is more biased towards the VBM, which means the formation of a *p*-type Schottky contact with the lateral hole SBH of 0.34 eV in ML SiAs FET with a graphene electrode. The Fermi levels are more biased towards the CBM, which means the formation of *n*-type Schottky contacts with the lateral electron SBHs of 0.28, 0.40, and 0.45 eV in ML SiAs FETs with Ag, Au, and Cu electrodes, respectively. The Fermi level moves down into the VBM of ML SiAs to form a *p*-type Ohmic contact with a V_2_CO_2_ electrode. The predicted contact type of the ML SiAs/Au interface is consistent with that measured in the experiment [21]. The intact ML SiAs is used without defects and grain boundaries in the calculation. However, inducing defects and grain boundaries for the devices in experiments is unavoidable. The defects and grain boundaries may be experimentally decreased by using the approaches, i.e., constructing vdW metal-2D semiconductor junctions [56] and applying a protective layer (such as *h*-BN) to the 2D material [57,58]. For example, vdW metal-2D MoS_2_ contacts are realized in the previous works [56,59]. 

The transport band gap is calculated by the sum of the lateral hole and electron SBHs, i.e., $E_{g,a}^{\mathrm{trans}}\;=\;\Phi_{L,a}^{e}{+\Phi }_{L,a}^{h}$. When V_2_CO_2_, Au, Ag, Cu, and graphene act as electrodes, the transport band gaps ($E_{g,a}^{\mathrm{trans}}$) are 1.67, 1.68, 1.70, 1.80, and 1.85 eV, respectively, which are consistent with the band gap of the pristine ML SiAs. As with the vertical contact, the lateral contact type and polarity along the *b* direction (from LDDOS in Figure 6) are the same as those along the *a* direction, except that the SBH is slightly different. The lateral *p*-type Schottky contact with the barrier height ($\Phi_{L,b}^{h}$) of 0.28 eV is discovered in ML SiAs FET with a graphene electrode, while the lateral *n*-type Schottky contacts are discovered with the barrier height ($\Phi_{L,b}^{e}$) of 0.27, 0.33, and 0.47 eV in ML SiAs FETs with Ag, Cu, and Au electrodes, respectively. The *p*-type Ohmic contact is discovered in ML SiAs FET with a V_2_CO_2_ electrode. 

**Figure 7:**
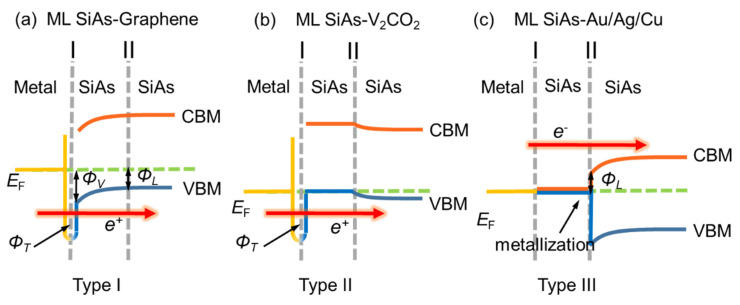
Three band patterns of ML SiAs FET based on the quantum transport calculation. Gray dashed lines represent the interface I and the interface II. $\Phi_{T}$, $\Phi_{V}$, and $\Phi_{L}$ represent tunneling barrier, vertical, and lateral Schottky barriers, respectively.

According to the barriers at these two interfaces, three types (type I, type II, and type III) of band patterns are identified in the explored ML SiAs FETs, as shown in Figure 7. In the type I, i.e., graphene as an electrode, there is a tunneling barrier with the tunneling probability of 99.48%, and vertical and lateral hole Schottky barriers with heights of 0.75 (0.65) eV and 0.34 (0.28) eV along the *a* (*b*) direction, respectively. In the type II, i.e., V_2_CO_2_ as an electrode, there is only a very small tunneling barrier with a tunneling probability of 95.65%, and a *p*-type Ohmic contact is discovered at both the vertical and lateral interfaces. In type III, i.e., Ag, Au, and Cu as electrodes, there are only *n*-type lateral electron Schottky barriers with heights of 0.28 (0.27), 0.40 (0.47), and 0.45 (0.33) eV along the *a* (*b*) direction, respectively. The tunneling probability at the ML SiAs/V_2_CO_2_ interfaces is 95.65%; therefore, V_2_CO_2_ will be the best choice for electrodes of ML SiAs transistors in the examined metals. 

**Figure 8:**
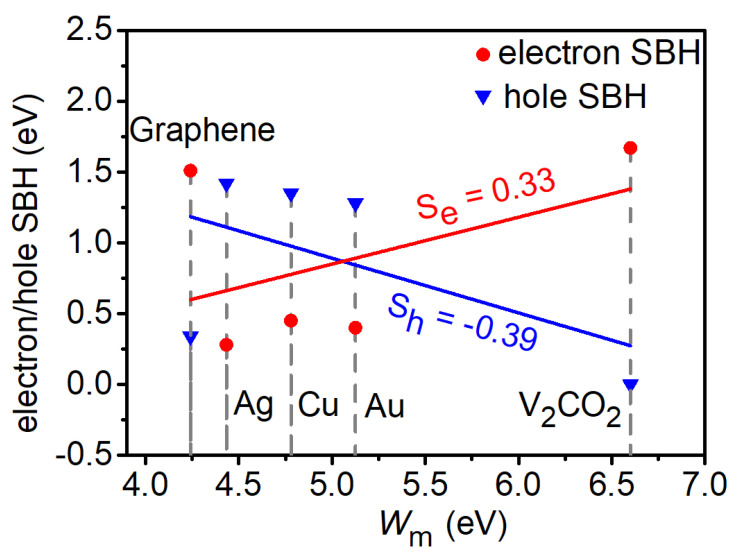
The plot of the relative lateral electron/hole SBH with the transport direction along the *a* direction versus the work function values of the electrodes.

A strong FLP effect always appears in 2D semiconductor FETs, such as in the ML TMDs FETs [34,60], and the FLP effect can make the Schottky barrier unadjustable by metals. Although Schottky barriers are induced by the metal-induced gap states in 2D TMDs [61,62], there are, in contrast, only a small amount of metal-induced gap states observed in the ML SiAs/Cu interface along the *a* direction, as shown in Figure 5f. To evaluate the degree of the FLP effect in ML SiAs FET, the variation of the lateral electron/hole SBH along the *a* direction with the work function of the electrode is shown in Figure 8. The level of FLP is determined by the electron/hole pinning factor $S_{e/h}$, which is established by the formula:

(4) $$S_{e/h}=\frac{d\Phi_{L,a}^{e/h}\;}{dW_{m}}$$

where $\Phi_{L,a}^{e/h}$ is the lateral electron/hole SBH and *W*_m_ is the work function of the pristine electrode [32]. The pinning factor changes from *S*_e/h_ = 0 for a completely pinned contact to *S*_e/h_ = 1/−1 for an unpinned contact (Schottky limit). The pinning factor *S*_e/h_ of ML SiAs is found to be 0.33/−0.39, indicating that there is a strong FLP in this system, but its FLP is smaller than that of MoS_2_ (*S* = 0.11) and MoTe_2_ (*S* = −0.07) [32]. The creation of Schottky barriers and FLP in ML SiAs FETs is mainly from the interaction between the electrode and the channel ML SiAs considered in the quantum transport simulation.

## 4. Conclusions

In this work, the interfacial properties in anisotropic ML SiAs FETs are comprehensively studied with electrodes (graphene, V_2_CO_2_, Au, Ag, and Cu) by using ab initio electronic structure calculations and quantum transport simulation. ML SiAs forms a weak interaction with graphene and V_2_CO_2_, while it forms a strong interaction with bulk metals (Au, Ag, and Cu). Although ML SiAs has strong anisotropy, the latter is not reflected in the contact property. The *p*-type vertical and lateral Schottky barriers are found with the SBH of 0.75 (0.65) eV and 0.34 (0.28) eV along the *a* (*b*) direction, in ML SiAs with graphene, respectively. The lateral *n*-type Schottky barriers are found with SBH of 0.28 (0.27), 0.40 (0.47) and 0.45 (0.33) eV along the *a* (*b*) direction with Ag, Au and Cu, respectively. In particular, ideal Ohmic contacts are formed at both vertical and lateral interfaces with V_2_CO_2_; thus, it can be an ideal electrode for ML SiAs FETs. This work not only provides a comprehensive study of the interface characteristics in ML GeAs FETs, but also gives a theoretical guide for the electrode screening of ML SiAs devices in experiments. 

## Figures and Tables

**Table 1:** **Calculated contact properties of the interfacial systems.** $\bar{E}$ is the average lattice parameter mismatch between ML SiAs and the electrode. $D_{z}$ is the average distance between ML SiAs and electrode. $E_{b}$ is the binding energy between electrode and ML SiAs. $W_{m}$ is the work function of the pristine electrode surface. $\Phi_{L,a}^{e}$ ($\Phi_{L,a}^{h}$) and $\Phi_{L,b}^{e}$ ($\Phi_{L,b}^{h}$) are the lateral electron (hole) SBH along the *a* and the *b* directions in the ML SiAs FETs, respectively, which are obtained by quantum transport simulation. $E_{g,a}^{\mathrm{trans}}$ and $E_{g,b}^{\mathrm{trans}}$ are the transport gap along the *a* and *b* directions, respectively. The work function of ML SiAs is 5.32 eV.

	Graphene	V_2_CO_2_	Au	Ag	Cu
$\bar{\varepsilon }\;(\%)$	0.15	2.81	2.84	2.84	2.39
$d_{z}\;(Å)$	3.29	2.55	2.31	2.30	2.07
$E_{b}\;(\mathrm{eV})$	0.32	0.46	0.59	0.57	0.74
$W_{m}\;(\mathrm{eV})$	4.24	6.60	5.13	4.43	4.78
$\Phi_{L,a}^{e}\;(\mathrm{eV})$	1.51	1.67	0.40	0.28	0.45
$\Phi_{L,a}^{h}\;(\mathrm{eV})$	0.34	0	1.28	1.42	1.35
$\Phi_{L,b}^{e}\;(\mathrm{eV})$	1.56	1.67	0.47	0.27	0.33
$\Phi_{L,b}^{h}\;(\mathrm{eV})$	0.28	0	1.36	1.55	1.49
$E_{g,a}^{\mathrm{trans}}\;(\mathrm{eV})$	1.85	1.67	1.68	1.70	1.80
$E_{g,b}^{\mathrm{trans}}\;(\mathrm{eV})$	1.84	1.67	1.83	1.82	1.82

## Data Availability

Data supporting the results of this study are available from the corresponding authors upon reasonable request.

## Supplementary Materials

The following supporting information can be downloaded at: https://www.mdpi.com/article/10.3390/nano14030238/s1, Figure S1: Side views of the non-optimized structures of ML SiAs on graphene, V_2_CO_2_, Au, Ag, and Cu surfaces.
