# Supplementary material for: Interfacial Properties of Anisotropic Monolayer SiAs Transistors
Source: Nanomaterials (Basel). 2024 Jan 23;14(3):238. doi: 10.3390/nano14030238 (PMC10856446; doi:10.3390/nano14030238)
Supplement: Supplementary file 1 [file nanomaterials-14-00238-s001.zip › nanomaterials-2835334-supplementary.pdf]

Supporting information

Interfacial Properties of Anisotropic Monolayer SiAs Transistors

Feihu Zou, [1,†] Yao Cong, [2,†] Weiqi Song, [1] Haosong Liu, [2] Yanan Li, [2] Yifan Zhu, [2] Yue Zhao, [1] Yuanyuan Pan [1,*], Qiang Li [1,*]

[1]College of Physics, Qingdao University, Qingdao 266071, China
[2]State Key Laboratory of Heavy Oil Processing, Institute of New Energy, College of Chemistry and Chemical Engineering, China University of Petroleum (East China), Qingdao 266580, China
[†]These authors contributed equally to this work.
*E-mail: panyy@qdu.edu.cn; liqiang@qdu.edu.cn

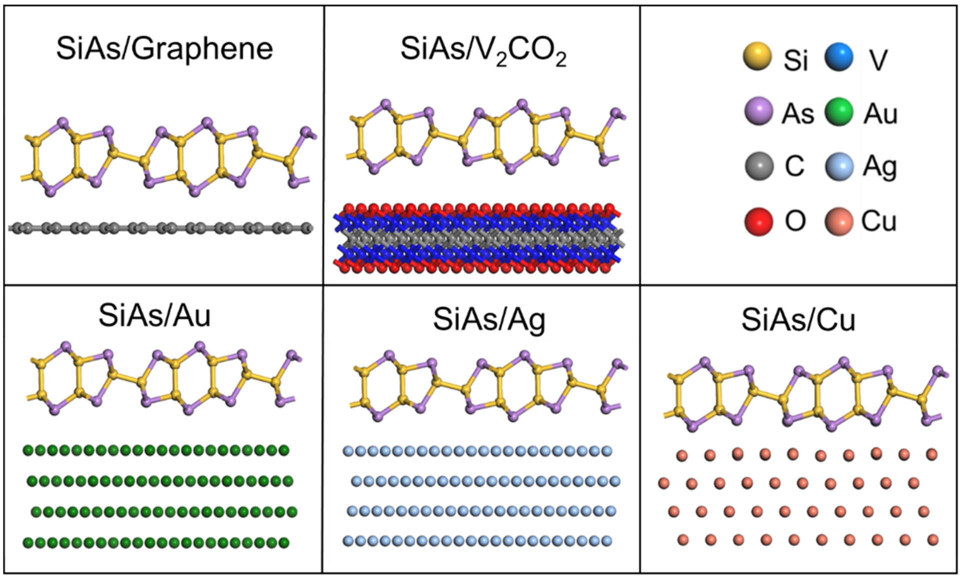

Figure S1. Side views of the non-optimized structures of ML SiAs on graphene, V_2CO_2, Au, Ag, and Cu surfaces.
